# Th17-associated cytokine gene hypomethylation reflects epigenetic dysregulation in graves’ disease

**DOI:** 10.3389/fimmu.2025.1635883

**Published:** 2025-09-16

**Authors:** Yanfei Jiang, Kaida Mu, Zhaowei Huang, Xinwei Zhang, Yalin Wang, Wenyu Xu, Ronghua Song, Jinan Zhang

**Affiliations:** ^1^ Graduate School, Shanghai University of Traditional Chinese Medicine, Shanghai, China; ^2^ Department of Endocrinology & Rheumatology, Shanghai University of Medicine & Health Sciences Affiliated Zhoupu Hospital, Shanghai, China

**Keywords:** Graves’ disease, DNA methylation, IL17, IL21, IL22, epigenetic biomarkers

## Abstract

**Introduction:**

Graves’ disease (GD) is an organ-specific autoimmune disorder characterized by the presence of thyroid-stimulating hormone receptor autoantibodies (TRAb), leading to hyperthyroidism. While genetic and environmental factors contribute to GD pathogenesis, the role of epigenetic mechanisms, particularly in regulating Th17-associated cytokines, remains poorly understood.

**Methods:**

This study aimed to characterize the promoter methylation profiles of IL17, IL21, and IL22 in GD patients, evaluate their diagnostic potential, and explore correlations with clinical parameters. Targeted bisulfite sequencing was performed on peripheral blood mononuclear cells from 60 GD patients, including newly diagnosed and refractory individuals, and 60 matched healthy controls.

**Results:**

Significant hypomethylation at IL17, IL21, and IL22 promoter regions was observed in GD patients compared with controls (P = 2.5 × 10⁻⁷), with partial methylation restoration in refractory cases. Four specific CpG sites were identified as potential biomarkers, demonstrating good diagnostic performance with area under the curve (AUC) values exceeding 0.7, including chr4_123542549_R (AUC = 0.754) and chr12_68647247_R (AUC = 0.752). These sites were associated with elevated TRAb (OR = 4.00, P = 0.02) and FT4 levels (OR = 0.29, P = 0.02), respectively.

**Discussion:**

Our findings highlight Th17-related epigenetic dysregulation as a key feature of GD and support the potential of methylation markers for diagnostic and therapeutic monitoring applications.

## 1 Introduction

Graves’ disease (GD) is a prototypical organ-specific autoimmune thyroid disorder characterized by hyperthyroidism mediated by thyroid-stimulating hormone receptor autoantibodies (TRAb), along with thyroid follicular epithelial hyperplasia and lymphocytic infiltration of the gland (1, 2). Its pathogenesis involves a complex interplay between genetic predisposition and environmental triggers. Genetic variants in immune-regulatory genes such as HLA, CTLA4, and PTPN22 are strongly associated with increased susceptibility to GD (3, 4), while environmental exposures are recognized as critical contributors to disease initiation and progression (5, 6).

Emerging evidence has shown elevated frequencies of peripheral Th17 cells in GD patients, correlating positively with TRAb titers (7). Th17-related cytokines—interleukin (IL)-17, IL-21, and IL-22—play distinct and complementary roles in the pathogenesis of autoimmune diseases. IL-17 promotes the recruitment of inflammatory cells and tissue remodeling; IL-21 facilitates B cell differentiation and autoantibody production; and IL-22 modulates epithelial barrier integrity and influences the local thyroid immune microenvironment (8–10). While their immunological functions in GD have been well established, the epigenetic regulation of these cytokines—particularly DNA methylation of their promoter regions—remains poorly defined (11, 12).

DNA methylation, a key epigenetic mechanism involving the addition of methyl groups to CpG dinucleotides, is essential for the regulation of gene expression and has been implicated in the pathogenesis of multiple autoimmune diseases (13–15). Hypomethylation of immune-related genes can result in aberrant T and B lymphocyte activation, aggravating inflammatory responses and promoting disease progression (16, 17). Previous studies suggest that methylation changes in cytokine genes may modulate their transcription and contribute to immune dysregulation in GD (18). Furthermore, environmental factors such as endocrine-disrupting chemicals may exacerbate GD by disrupting thyroid hormone signaling and interfering with epigenetic regulation (19). A comprehensive understanding of the interplay among genetic susceptibility, environmental factors, and epigenetic modifications is therefore essential for elucidating the molecular mechanisms underlying GD and identifying novel therapeutic targets (20).

In this study, we employed targeted bisulfite sequencing to assess the DNA methylation status of the promoter regions of IL17, IL21, and IL22 in peripheral blood mononuclear cells (PBMCs) from GD patients (21). Using machine learning algorithms, we developed a CpG-based diagnostic model and, for the first time, compared methylation profiles between newly diagnosed GD (NGD) and refractory GD (RGD) cases (22). By correlating methylation patterns with clinical parameters such as TRAb and FT4, we aimed to elucidate the role of Th17-associated epigenetic dysregulation in GD progression and identify potential biomarkers for disease diagnosis and therapeutic monitoring.

## 2 Materials & methods

### 2.1 Study population

This study was conducted at Zhoupu Hospital, Shanghai, China, between August 2023 and January 2025. A total of 120 Han Chinese individuals were enrolled, including 60 patients diagnosed with GD and 60 age- and sex-matched healthy controls. Participants were recruited from the Department of Endocrinology and the Physical Examination Center. Baseline demographic and clinical characteristics are summarized in **Table 1**.

**Table 1:** Characteristics of patients with GD and controls.

Characteristic	GD (%)		Control (%)	p
	NGD (%)	RGD (%)	NC (%)	
Number	35	25	60	–
Sex				0.064
Female	35	21	55	
Male	0	4	5	
Age	36.46±13.49	37.20±12.85	38.25±1.60	0.499
Age of onset	36.46±13.49	33.12±12.94	–	0.324
<=18y	1	2		
>18y	34	23		
Thyroid size				0.676
Normal	22	16	–	
I	5	4	–	
II	6	5	–	
III	2	0	–	
Family history				0.764
(+)	2	3	–	
(-)	33	23	–	
Ophthalmopathy				0.937
(+)	3	3	–	
(-)	32	22	–	
Smoke				0.162
(+)	2	3	–	
(-)	31	28	–	
T3(1.21-3.01nmol/l)	6.38±2.37	4.69±2.60	–	–
T4(71.31-165.06nmol/l)	229.13±85.56	207.56±71.51	–	–
FT3(3.15-6.70pmol/l)	25.79±12.66	16.00±10.88	–	–
FT4(11.92-21.62pmol/l)	57.69±29.73	37.24±18.40	–	–
TSH(0.300-5.000uIU/l)	0.01±0.02	0.01±0.00	–	–
Tg(1.59-50.03ng/ml)	38.79±74.86	58.86±102.13	–	–
TgAb(<115.00IU/ml)	540.37±758.96	326.73±456.94	–	–
TPOAb(<34IU/ml)	346.57±287.12	747.61±420.35	–	–
TRAb(<1.50IU/l)	13.19±12.36	6.62±3.25	–	–
Drug				–
≥5mg/d	–	14	–	
<5mg/d	–	2	–	

Data are mean±SEM;GD Graves’ Disease, NGD Newly Diagnosed Hyperthyroidism, RGD Refractory Graves’ Disease, NC Normal Control, Normal No thyroid enlargement, thyroid is not palpable, or not visible on imaging, I Mild thyroid enlargement, palpable only when swallowing, or detectable by imaging, II Moderate thyroid enlargement, palpable at rest, and clearly visible on imaging, III Severe thyroid enlargement, visibly affecting the neck, often forming a visible goiter, and affecting swallowing and breathing, (+) indicates the presence of the condition (e.g., family history, ophthalmopathy, smoking). (-) indicates the absence of the condition, T3 Triiodothyronine, T4 Thyroxine, FT3 Free Triiodothyronine, FT4 Free Thyroxine, TSH Thyrotropin (Thyroid-Stimulating Hormone), Tg Thyroglobulin, TgAb Thyroglobulin Antibody, TPOAb Thyroid Peroxidase Antibody, TRAb TSH Receptor Antibody.

To investigate disease phenotypes, GD patients were further stratified into two subgroups: (1) NGD, defined as patients with first-time diagnosis of hyperthyroidism and no prior treatment; and (2) RGD, defined as patients with a disease duration >2 years, persistent or recurrent hyperthyroid symptoms, and positive TRAb levels (≥1.5 IU/L) despite standard treatment. Clinical data collected included age at onset, thyroid size, presence of ophthalmopathy, smoking status, and family history of hyperthyroidism (defined as having at least one first-degree relative affected). Drug history was recorded for methimazole (MMI), and patients were categorized according to their daily dosage (≥5 mg/day or <5 mg/day).The study was approved by the Ethics Committee of Shanghai Health Medical College (Approval No. 2023-C-123-E01), and written informed consent was obtained from all participants.

### 2.2 Thyroid function assessment and inclusion/exclusion criteria

GD diagnosis was based on clinical symptoms, laboratory findings, and thyroid ultrasonography. Fasting venous blood samples (10 mL, non-anticoagulated) were collected after ≥8 hours of fasting. Thyroid function was assessed using electrochemiluminescence (ECL) assays. Inclusion criteria for GD comprised: clinical manifestations of hyperthyroidism, suppressed serum TSH levels, positive TRAb, and ultrasonographic evidence of diffuse goiter.

Healthy controls were selected from individuals with normal physical and biochemical examination results and no history of chronic diseases. Control participants also provided fasting venous blood samples, and their thyroid peroxidase antibody (TPOAb) levels were assessed using ECL. Inclusion criteria for controls included TPOAb negativity and absence of a family history of autoimmune thyroid diseases (AITDs). Exclusion criteria for both groups included the presence of diffuse goiter or thyroid nodules >5 mm detected by ultrasonography, as well as a history or diagnosis of other autoimmune diseases.

### 2.3 DNA extraction

PBMCs were isolated from 5.0 mL of venous blood collected in EDTA-K2 tubes after overnight fasting. Genomic DNA was extracted using the Relaxgene DNA Isolation Kit (Tiangen Biotech, China) following the manufacturer’s instructions. DNA concentration and purity were assessed using a NanoDrop 2000 spectrophotometer (Thermo Scientific, USA). Only samples meeting the following quality criteria were included: concentration ≥20 ng/μL, OD260/280 ratio between 1.7–1.9, and OD260/230 ≥2.0.

### 2.4 DNA methylation analysis of IL17, IL21, and IL22

DNA methylation levels at the promoter regions of IL17, IL21, and IL22 genes were analyzed using the MethylTarget™ assay (Genesky Corporation, Shanghai, China). CpG-rich promoter regions (GC content >20%, length >200 bp) were selected, and two rounds of PCR were conducted per target gene. Methylation at each CpG site was quantified as the ratio of methylated to total cytosines. CpG site annotation was based on genomic coordinates. Primer sequences and PCR conditions are provided in **Table 2** and **Supplementary Table S1**.

**Table 2:** IL17, IL21, IL22 CpG site annotation.

IL17				IL21				IL22			
CpG Site	Strand	Location	DistanceToTSS	CpG Site	Strand	Location	DistanceToTSS	CpG Site	Strand	Location	DistanceToTSS
chr6_52049313_F	+	Promoter	-1872	chr4_123542199_R	–	Promoter	25	chr12_68647247_R	–	Promoter	34
chr6_52049431_F	+	Promoter	-1754	chr4_123542356_R	–	Promoter	-132	chr12_68647281_R	–	Promoter	0
chr6_52049562_F	+	Promoter	-1623	chr4_123542401_R	–	Promoter	-177	chr12_68647290_R	–	Promoter	-9
chr6_52049644_F	+	Promoter	-1541	chr4_123542549_R	–	Promoter	-325	chr12_68647352_R	–	Promoter	-1472
chr6_52050133_F	+	Promoter	-1383	chr4_123543093_R	–	Promoter	-869	chr12_68647357_R	–	Promoter	30
chr6_52050190_F	+	Promoter	-995	chr4_123543180_R	–	Promoter	-956	chr12_68647388_R	–	Promoter	-1
chr6_52050263_F	+	Promoter	-922	chr4_123543435_R	–	Promoter	-1463	chr12_68647499_R	–	Promoter	-112
chr6_52050401_F	+	Promoter	-1734	chr4_123543739_R	–	Promoter	-1515	chr12_68647575_R	–	Promoter	-188
chr6_52050597_F	+	Promoter	-588	chr4_123543922_R	–	Promoter	-1698	chr12_68647591_R	–	Promoter	-204
chr6_52050745_F	+	Promoter	-440	chr4_123543942_R	–	Promoter	-1718	chr12_68647735_R	–	Promoter	-348
chr6_52051062_F	+	Promoter	-123					chr12_68648357_R	–	Promoter	-912
chr6_52051103_F	+	Promoter	-82					chr12_68648359_R	–	Promoter	-1532
chr6_52051108_F	+	Promoter	-77					chr12_68648412_R	–	Promoter	-1481
chr6_52051113_F	+	Promoter	-72					chr12_68648630_R	–	Promoter	-1243
chr6_52051156_F	+	Promoter	-29					chr12_68648813_R	–	Promoter	-1426
chr6_52051162_F	+	Promoter	-23					chr12_68648921_R	–	Promoter	-1534
								chr12_68649043_R	–	Promoter	-1412

F forward primer, R reverse primer,+Sense strand,-Antisense strand.

### 2.5 Diagnostic model construction using machine learning

To identify potential diagnostic biomarkers, four supervised machine learning algorithms were applied: Random Forest (RF), Support Vector Machine (SVM), Generalized Linear Model (GLM), and eXtreme Gradient Boosting (XGBoost). All models were implemented in R using the caret package (v7.0-1) with standardized preprocessing including mean-centering and near-zero variance filtering (23). Model performance was evaluated using repeated stratified 5-fold cross-validation (3 repetitions), with performance metrics averaged across all folds.

Model interpretability was assessed using Shapley additive explanations (SHAP), implemented via the DALEX R package (v2.4.3), to evaluate the relative importance of each CpG site based on cooperative game theory (24). Diagnostic performance was quantified using receiver operating characteristic (ROC) curves generated by the pROC package (v1.18.5), and the area under the curve (AUC) was interpreted as follows: <0.6, non-informative; 0.6–0.7, suboptimal; 0.7–0.9, moderate to strong; >0.9, excellent discrimination (25).

### 2.6 Correlation between methylation and clinical parameters

To explore the relationship between methylation profiles and clinical features of GD, patients were stratified by age, age at onset, sex, disease subtype, family history, presence of ophthalmopathy, smoking status, and thyroid function indices. FT3 and FT4 levels were dichotomized at their median values. TSH was categorized as ≤0.001 vs. >0.001 mIU/L, and TRAb as ≤1.5 vs. >1.5 IU/L. Variables with >50% missing data (e.g., T3, T4, Tg) were excluded.

Expression data were log2-transformed using the formula log2(X+1). Univariate binary logistic regression was performed using the glm function in R to identify significant predictors. Variables with p < 0.1 were included in multivariate logistic regression to identify independent associations, with significance set at p < 0.05. Statistical modeling was conducted using the rms (v6.4.0) and ResourceSelection (v0.3.5) R packages.

### 2.7 Statistical analysis

Methylation data are presented as mean±standard error of the mean (SEM). Normally distributed continuous variables are shown as mean±standard deviation (SD), while non-normally distributed data are expressed as median with interquartile range (IQR). Group comparisons were conducted using independent samples t-tests or Mann–Whitney U tests, as appropriate. Correlations between methylation levels and clinical variables were evaluated using Spearman’s rank correlation coefficient. Categorical variables were analyzed using Chi-square or Fisher’s exact tests.

Univariate binary logistic regression was performed to evaluate associations between clinical features and methylation subgroups, and odds ratios (ORs) with 95% confidence intervals (CIs) were calculated. Variables with p < 0.1 in univariate analysis were included in multivariate logistic regression to identify independent predictors (p < 0.05). All statistical analyses, including data preprocessing, descriptive statistics, and regression modeling, were conducted using SPSS version 26.0 (IBM Corp., Armonk, NY, USA) and R version 4.3.3 (R Core Team, Vienna, Austria).

## 3 Results

### 3.1 Reduced methylation levels of IL17, IL21, and IL22 promoter regions in GD patients

To investigate DNA methylation alterations across different stages of Graves’ disease (GD), we enrolled 35 NGD patients, 25 RGD patients, and 60 NCs. PBMCs were isolated and subjected to DNA extraction. Following stringent quality control, promoter regions of the Th17-related cytokines IL17, IL21, and IL22 with GC content >20% were selected for methylation analysis.

**Figure 1:**
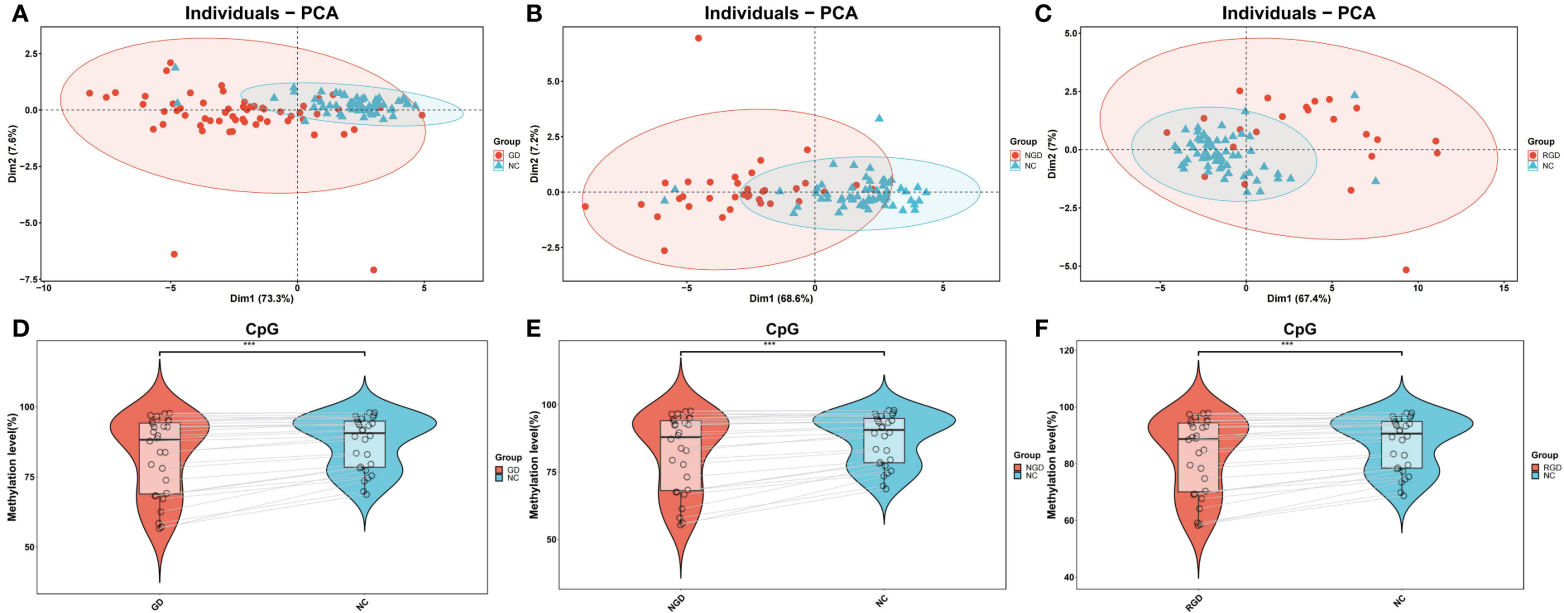
DNA methylation percentages in IL17, IL21, and IL22 gene promoters across study groups. **(A)** Principal component analysis (PCA) of DNA methylation levels in the IL17, IL21, and IL22 gene promoters between Graves’ disease (GD) patients and normal controls (NC). **(B)** PCA of methylation levels comparing newly diagnosed GD (NGD) patients and NC. **(C)** PCA of methylation levels comparing refractory GD (RGD) patients and NC. **(D)** Violin plots showing DNA methylation percentages in the IL17, IL21, and IL22 promoters between GD and NC groups. **(E)** Violin plots comparing methylation levels between NGD and NC groups. **(F)** Violin plots comparing methylation levels between RGD and NC groups. Abbreviations: GD, Graves’ disease; NGD, newly diagnosed Graves’ disease; RGD, refractory Graves’ disease; NC, normal control; PCA, principal component analysis. *p < 0.05, **p < 0.01, ***p < 0.001, ns, not significant (p > 0.05).

Compared to NCs, the majority of CpG sites in GD patients exhibited significantly reduced methylation levels, with high intra-group consistency (**Supplementary Figures S1A-F**). Principal component analysis (PCA) of methylation profiles clearly distinguished GD patients from controls (**Figures 1A–C**), and revealed a significant overall decrease in methylation levels in GD patients (P=2.5 × 10^-7^, **Figure 1D**). Both NGD (P=1.4 × 10^-7^) and RGD (P=6.6 × 10^-7^) groups showed significantly lower methylation compared to NCs, with NGD exhibiting the most pronounced hypomethylation, suggesting stage-specific methylation dynamics (**Figures 1E–F**).

**Figure 2:**
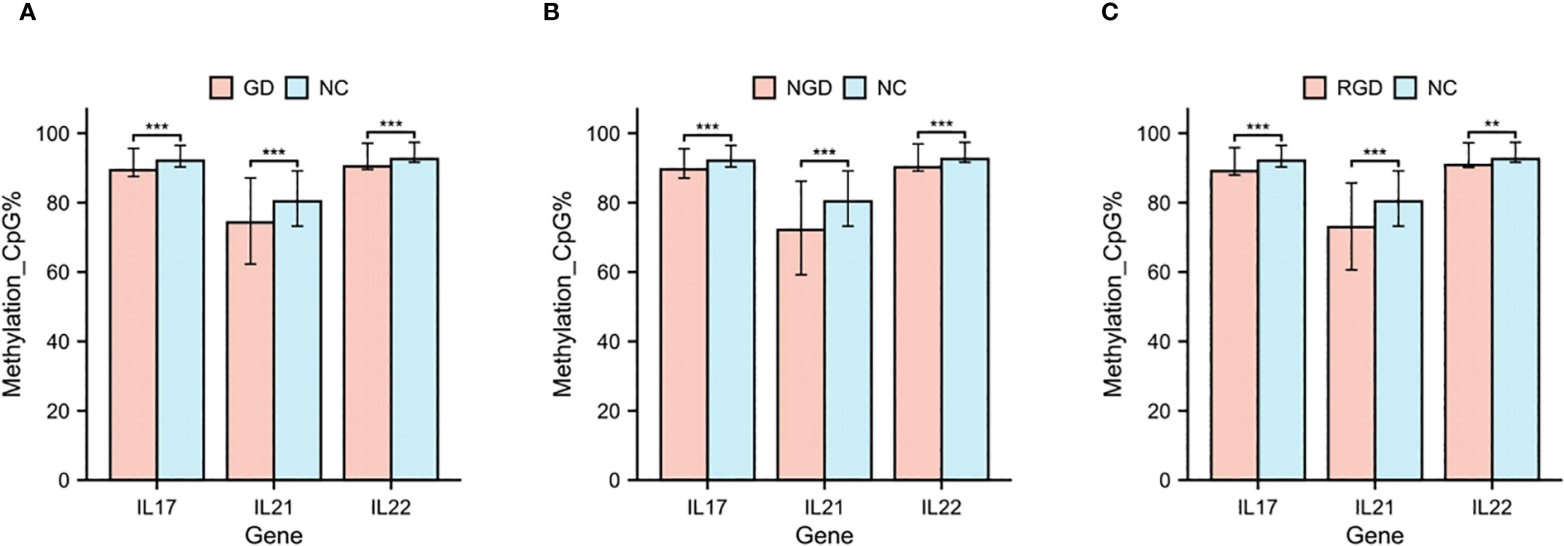
DNA methylation percentages in IL17, IL21, and IL22 gene promoters in each study group. **(A)** DNA methylation percentages in IL17, IL21, and IL22 gene promoters in GD patients and NC. **(B)** DNA methylation percentages in IL17, IL21, and IL22 gene promoters in NGD and NC. **(C)** DNA methylation percentages in IL17, IL21, and IL22 gene promoters in RGD and NC. GD, Graves’ Disease; NGD, Newly Diagnosed Graves’ Disease; RGD, Refractory Graves’ Disease; NC, Normal Control. *p < 0.05, **p < 0.01, ***p < 0.001, ns, p > 0.05.

We further assessed gene-specific methylation of the IL17, IL21, and IL22 promoter regions. All three genes exhibited significantly lower promoter methylation levels in GD patients relative to NCs (p < 0.05, **Figure 2A**). Similar reductions were observed in both NGD and RGD subgroups (p < 0.05, **Figures 2B, C**).

**Figure 3:**
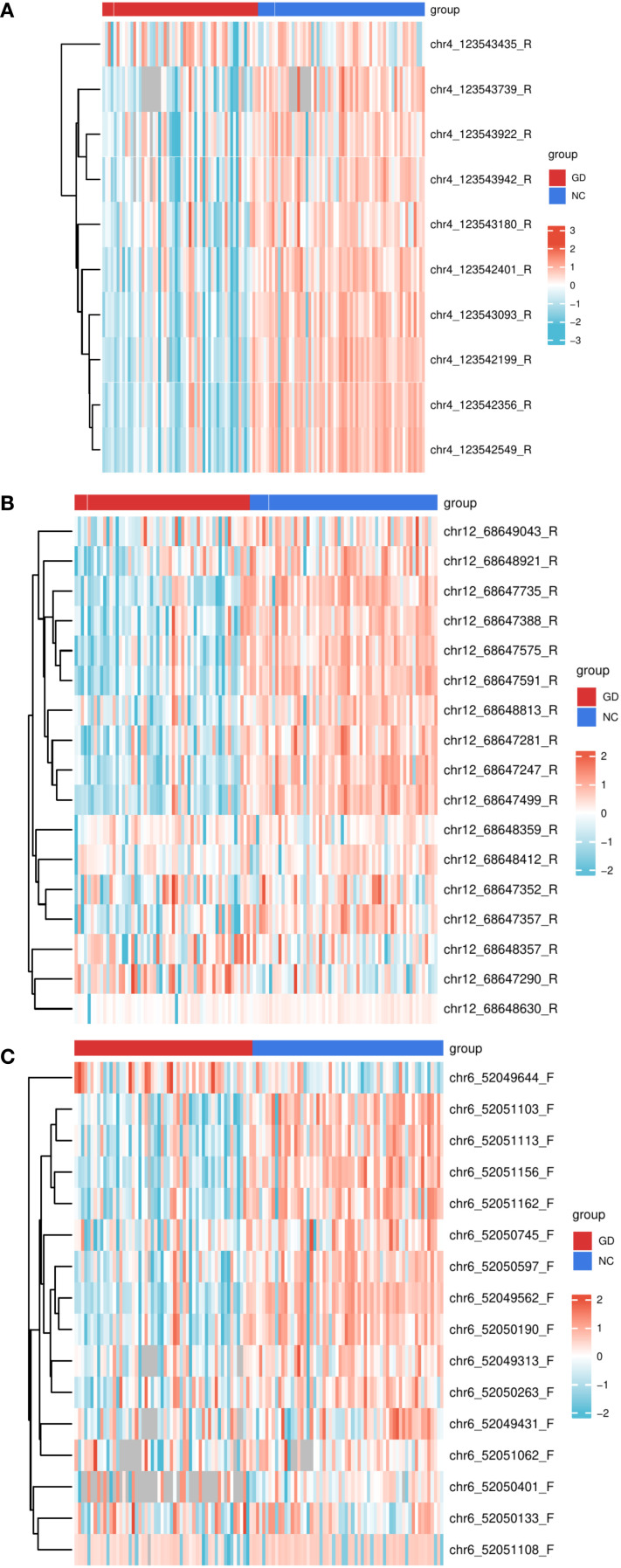
Heatmaps of DNA methylation levels in the promoter regions of IL17, IL21, and IL22 genes across study groups. **(A)** Heatmap depicting DNA methylation percentages at individual CpG sites within the IL17 gene promoter in patients with Graves’ disease (GD) compared to normal controls (NC). **(B)** Heatmap depicting DNA methylation percentages at individual CpG sites within the IL21 gene promoter in GD patients versus NC. **(C)** Heatmap depicting DNA methylation percentages at individual CpG sites within the IL22 gene promoter in GD patients versus NC. Each color gradient represents the relative methylation level at each CpG site, allowing for visualization of methylation patterns across groups. GD, Graves’ Disease; NC, Normal Control.

**Figure 4:**
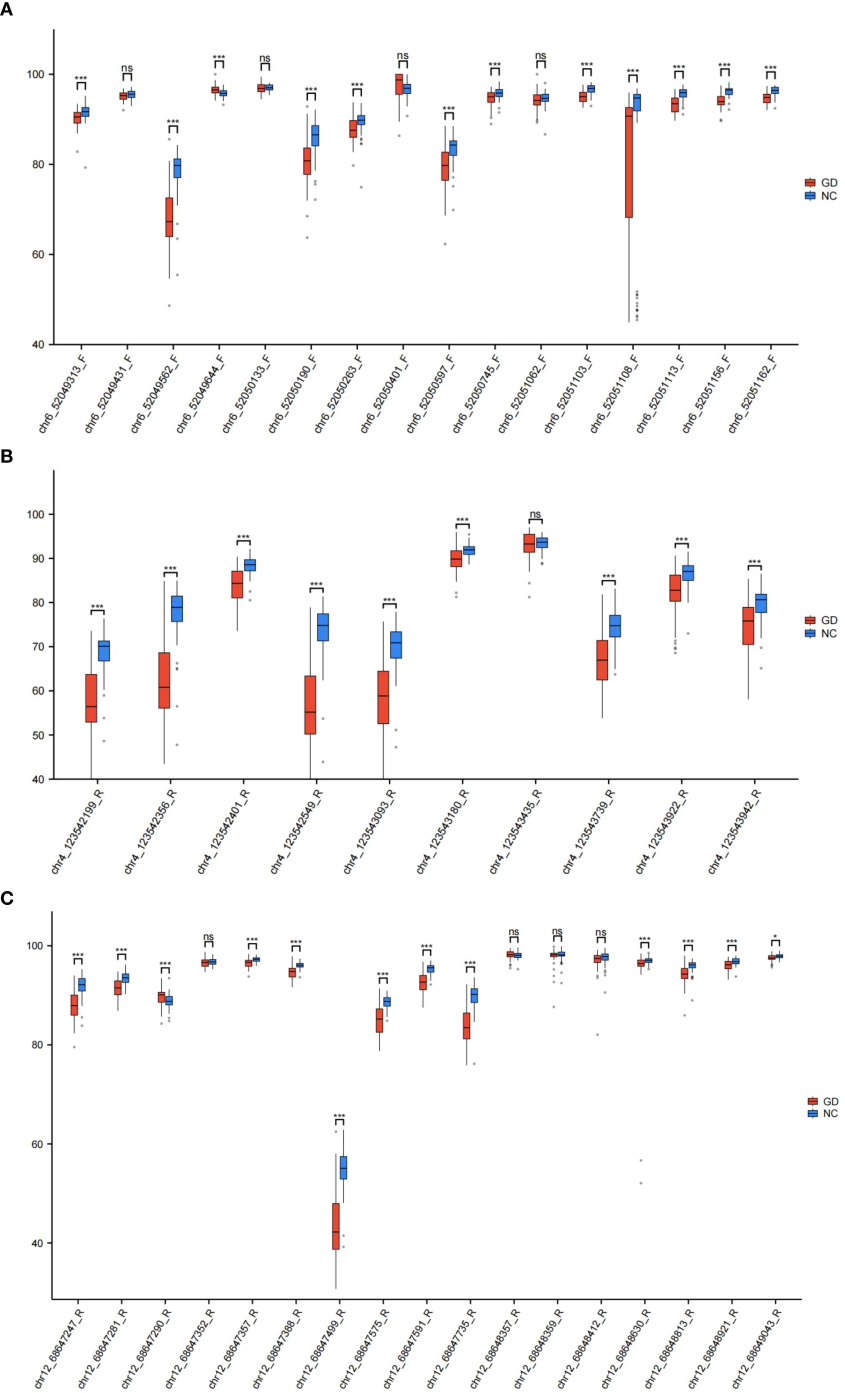
Box plots of DNA methylation levels in the promoter regions of IL17, IL21, and IL22 genes between study groups. **(A)** Box plot illustrating the distribution of DNA methylation percentages at CpG sites within the IL17 gene promoter in GD patients compared to NC. **(B)** Box plot illustrating the distribution of DNA methylation percentages at CpG sites within the IL21 gene promoter in GD patients versus NC. **(C)** Box plot illustrating the distribution of DNA methylation percentages at CpG sites within the IL22 gene promoter in GD patients versus NC. Data are presented as median with interquartile range; statistical comparisons highlight group-specific differences in methylation levels. GD, Graves’ Disease; NC, Normal Control. *p < 0.05, **p < 0.01, ***p < 0.001, ns, p > 0.05.

To delineate CpG site-specific methylation patterns, we analyzed promoter regions >200 bp in length, containing 16 CpG sites for IL17, 10 for IL21, and 17 for IL22 (**Supplementary Table S2**). The methylation levels of CpG sites for each gene are shown in **Figures 3A–C**. Site-level methylation analyses demonstrated significant hypomethylation in GD patients at 12 CpG sites in IL17 (p < 0.05, **Figure 4A**), 9 sites in IL21 (**Figure 4B**), and 13 sites in IL22 (**Figure 4C**).

### 3.2 Identification of diagnostic CpG Sites in IL17, IL21, and IL22 promoter regions using machine learning

**Figure 5:**
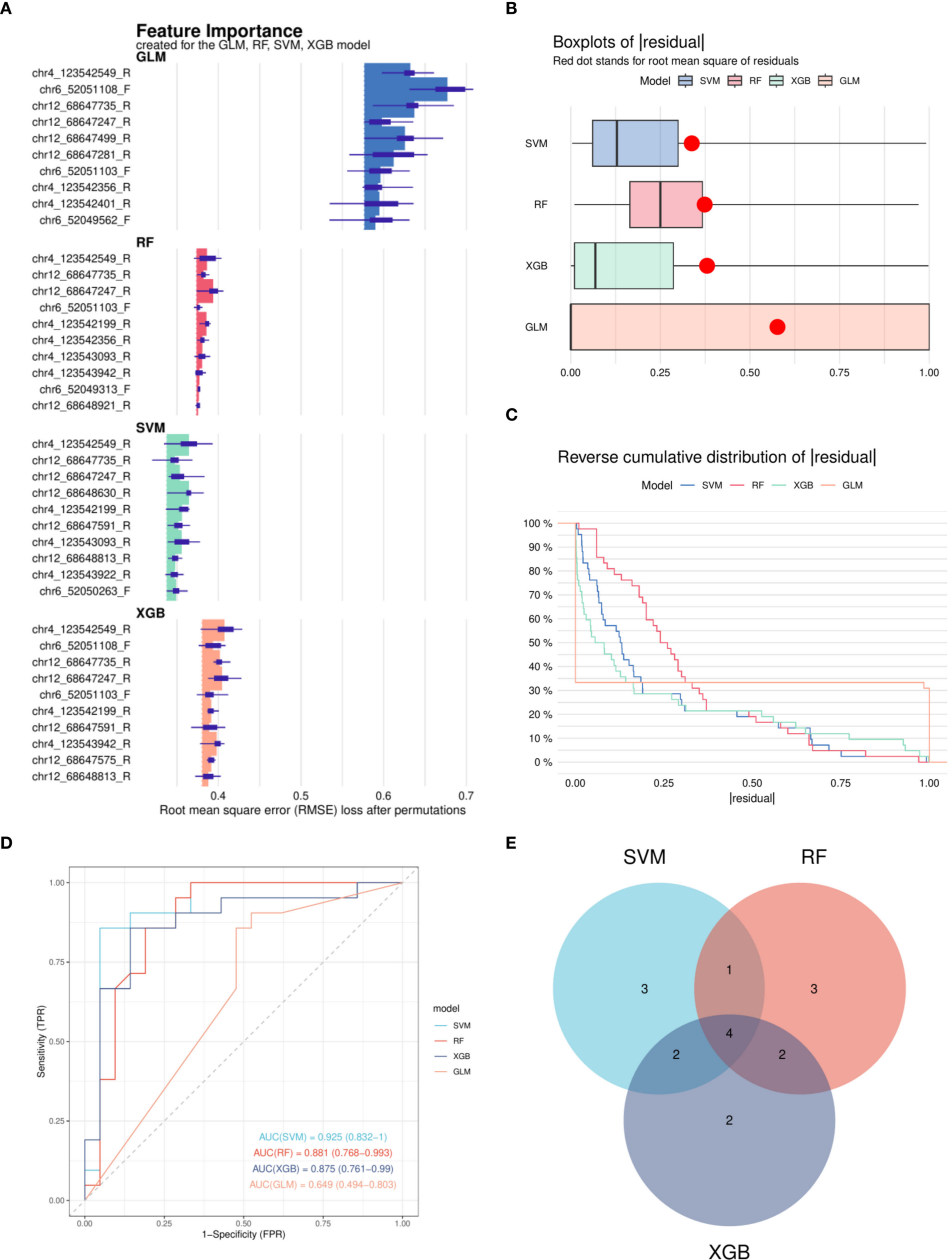
Construction and performance evaluation of machine learning models for DNA methylation-based classification. **(A)** Feature importance rankings derived from four machine learning models: Random Forest (RF), Support Vector Machine (SVM), Generalized Linear Model (GLM), and eXtreme Gradient Boosting (XGB). **(B)** Cumulative residual distribution curves for each model, indicating overall model fit. **(C)** Box plots of residuals for the four models; red dots represent the Root Mean Square Error (RMSE) for each model. **(D)** Receiver Operating Characteristic (ROC) curves generated from the test set for all four models; SVM, RF, and XGB models achieved area under the curve (AUC) values exceeding 0.7. **(E)** Venn diagram illustrating the overlap of important features identified by the SVM, RF, and XGB models. RF, Random Forest; SVM, Support Vector Machine; GLM, Generalized Linear Model; XGB, eXtreme Gradient Boosting; RMSE, Root Mean Square Error; ROC, Receiver Operating Characteristic; AUC, Area Under the Curve.

After excluding CpG sites without statistically significant methylation differences, the remaining CpG sites in the promoter regions of IL17, IL21, and IL22 were used to develop predictive models. Four machine learning algorithms were applied: RF, SVM, GLM, and XGB. Among them, the RF, SVM, and XGB models demonstrated superior performance in terms of lower residual distributions and higher classification accuracy based on ROC curve analysis (**Figures 5A–C**). Thus, these three models were selected for downstream analyses.

Four CpG sites—chr4_123542199_R, chr4_123542549_R, chr12_68647247_R, and chr12_68647735_R—were consistently identified by the three models and prioritized for further evaluation (**Figures 5D, E**).

### 3.3 Diagnostic evaluation of hypomethylated CpG sites

**Figure 6:**
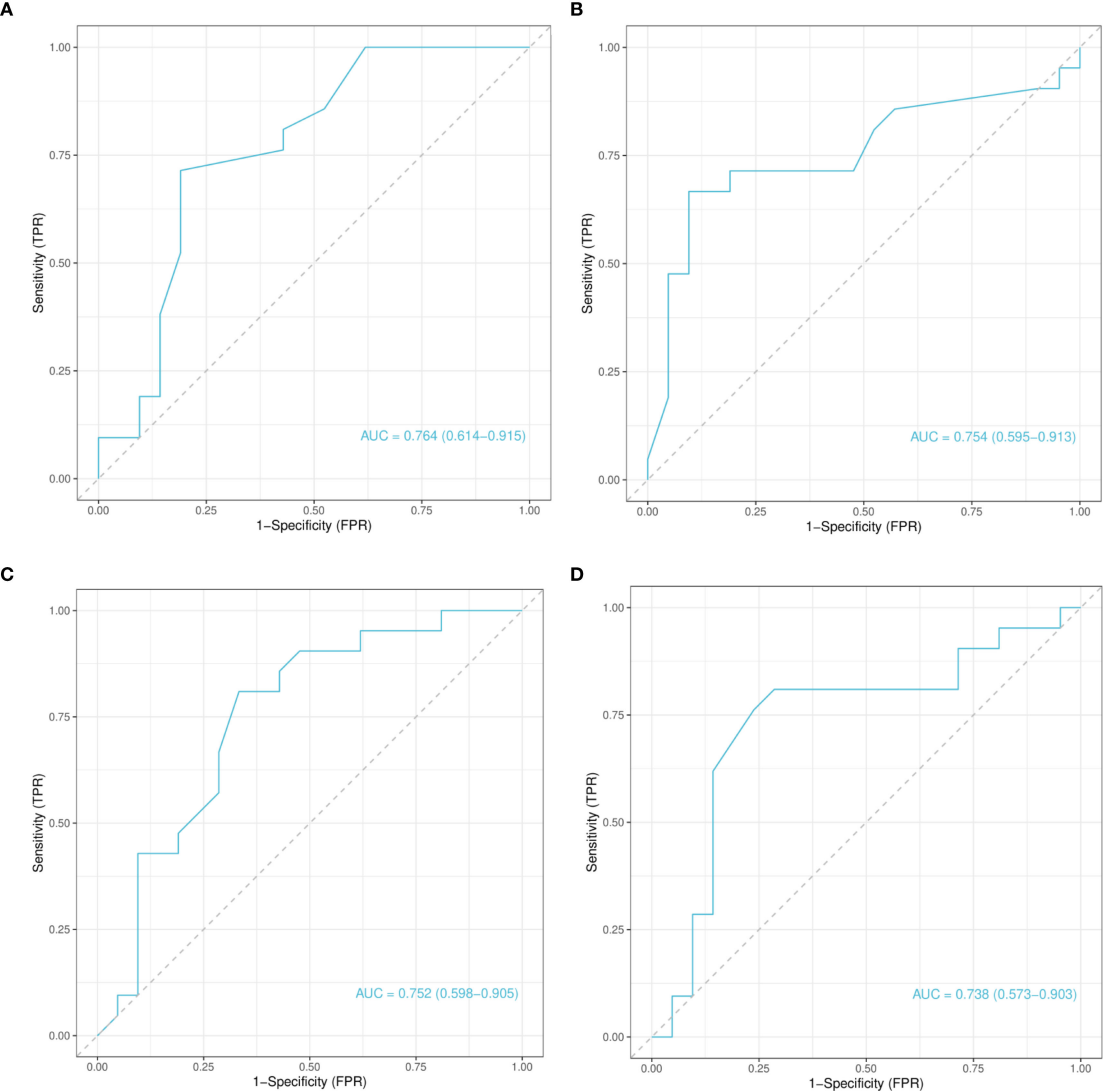
Receiver operating characteristic curve analysis of selected CpG Loci based on test set predictions. **(A)** Receiver Operating Characteristic (ROC) curve for the chr4_123542199_R locus. **(B)** ROC curve for the chr4_123542549_R locus. **(C)** ROC curve for the chr12_68647247_R locus. **(D)** ROC curve for the chr12_68647735_R locus. Each panel shows the classification performance of the corresponding CpG locus, with AUC values indicating diagnostic potential. ROC, Receiver Operating Characteristic; AUC, Area Under the Curve.

The four CpG sites identified through model-based feature importance were subjected to ROC curve analysis to assess their diagnostic utility (**Figure 6**). All four CpG sites demonstrated AUC values >0.7, indicating moderate diagnostic performance. Specifically, the AUC values for chr4_123542199_R, chr4_123542549_R, chr12_68647247_R, and chr12_68647735_R were 0.764, 0.754, 0.752, and 0.738, respectively (**Figures 6A–D**). These results suggest that hypomethylation at these sites may serve as potential biomarkers for GD diagnosis.

### 3.4 Clinical relevance of methylation alterations

**Figure 7:**
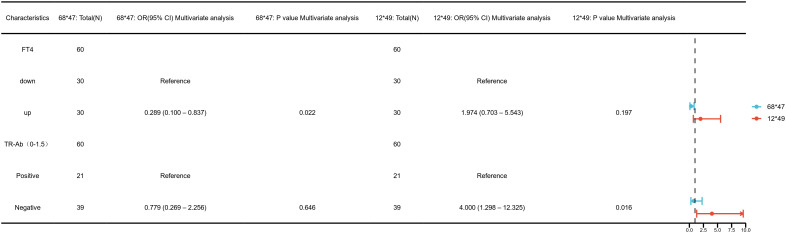
Multivariate logistic regression analysis of clinical associations at key DNA methylation sites— chr4_123542549_R (12*49), chr12_68647247_R (68*47).

Clinical variable distributions are presented in **Supplementary Table S4**. To investigate the clinical relevance of the four identified CpG sites, univariate logistic regression analyses were performed (**Supplementary Figure S2**). Hypomethylation at chr4_123542549_R was significantly associated with elevated TRAb levels (OR=4.00, 95% CI: 1.30–12.33, p=0.02), and this association remained significant in multivariate analysis (**Figure 7**; **Supplementary Table S3**). These findings suggest that demethylation at this site may influence TRAb production or activity, potentially contributing to disease pathogenesis and reflecting disease severity.

Furthermore, both univariate and multivariate analyses revealed a significant association between hypomethylation at chr12_68647247_R and increased FT4 levels (OR=0.29, 95% CI: 0.10–0.84, p=0.02, **Figure 7**; **Supplementary Table S3**). This indicates a possible regulatory role for this site in thyroid hormone synthesis or metabolism, suggesting its utility as a marker of thyroid functional status in GD.

No significant associations were observed between methylation levels at chr4_123542199_R or chr12_68647735_R and clinical parameters (p > 0.05, **Supplementary Figure S2**).

## 4 Discussion

In recent years, DNA methylation has been widely recognized as a key epigenetic mechanism involved in maintaining physiological homeostasis and regulating gene expression under pathological conditions, including autoimmune diseases (26, 27). GD, a prototypical autoimmune thyroid disorder, has been associated with aberrant methylation patterns of immune-related genes, such as IL10 (28). In this study, we focused on the DNA methylation status of the promoter regions of IL-17, IL-21, and IL-22 in PBMCs from patients with GD. By integrating machine learning models, we identified hypomethylated CpG sites significantly associated with GD, and found strong correlations between these epigenetic alterations and clinical indicators such as TRAb and FT4. These findings are consistent with previous reports emphasizing the role of Th17 cells in GD-related immune dysregulation and provide new insights into the epigenetic regulation of Th17 cell function (29, 30).

We found that the promoter regions of Th17-related cytokines exhibited significantly lower methylation levels in patients with GD compared to healthy controls. This suggests that epigenetic mechanisms may contribute to GD pathogenesis and highlights a previously underexplored dimension of Th17 cell regulation in this context. Notably, aberrant methylation patterns of IL-17, IL-21, and IL-22 are not exclusive to GD; similar patterns have been reported in other Th17-mediated autoimmune diseases, including rheumatoid arthritis, psoriasis, and Sjögren’s syndrome (31–34). Hypomethylation in these loci may enhance transcription factor binding, such as RORγt, or modulate IL-23/IL-23R signaling, or destabilize repressive chromatin complexes, thereby facilitating the expression of these pro-inflammatory cytokines (35–38). These observations support the existence of a shared epigenetic dysregulation of Th17-associated genes across various autoimmune conditions.

Previous studies, including our own, have demonstrated increased proportions of Th17 cells in patients with GD, with elevated expression levels of IL-17, IL-21, and IL-22 correlating positively with disease activity (39–42). These cytokines are markedly upregulated in GD and are implicated in amplifying the pro-inflammatory response and contributing to thyroid tissue damage (43–45). While earlier research has primarily focused on phenotypic aspects of Th17 cell activation, our study underscores the pivotal role of epigenetic regulation in modulating Th17-related gene expression. By systematically correlating promoter methylation with transcriptional activity, we offer new insights into the epigenetic mechanisms underlying Th17 dysregulation in GD (46, 47).

GD is characterized by thyroid-specific organ involvement, in which aberrant expression of the thyroid-stimulating hormone receptor (TSHR) on follicular epithelial cells and its interaction with thyroid-stimulating hormone receptor antibodies (TRAb) may drive immune imbalance (48). However, our machine learning analysis revealed that hypomethylation at chr4:123542549_R (AUC > 0.7) exhibited higher sensitivity and specificity for GD identification compared to conventional antibody-based diagnostics such as TRAb, which has a false-negative rate of 10–15% (49). Additionally, the methylation level at chr12:68647247_R was significantly correlated with elevated FT4 levels. These findings suggest that aberrant methylation at specific loci may play a direct regulatory role in the immune imbalance and thyroid dysfunction associated with GD. By employing advanced machine learning techniques, this study uniquely identified GD-associated CpG sites with potential predictive value for disease risk, warranting further validation in larger cohorts (50).

Interestingly, in patients with RGD, partial modulation of methylation levels was observed after treatment; however, these levels remained lower than in healthy controls. This suggests the presence of an epigenetic “memory” that may sustain autoimmune activity and contribute to disease relapse, even during periods of clinical remission. Persistent hypomethylation of Th17 cytokine genes may maintain Th17 activation and predispose patients to recurrence. This finding offers new perspectives on the mechanisms underlying GD chronicity and relapse. Clinically, methylation status may reflect treatment response and serve as a biomarker for relapse risk. The incomplete restoration observed suggests that conventional antithyroid therapy (e.g., methimazole) may insufficiently reset epigenetic patterns, highlighting the need for interventions targeting immune regulation or epigenetic remodeling. Longitudinal monitoring of methylation profiles could aid early diagnosis, track disease trajectory, and identify patients at high risk of persistent or recurrent hyperthyroidism. Future research should employ single-cell methylome sequencing and functional assays to dissect cell type–specific methylation patterns and clarify their roles in modulating Th17 activity. Furthermore, dynamic monitoring of methylation changes in Th17 cytokine genes may inform treatment adjustments and support the development of early-stage immuno-epigenetic combination therapies (51).

In conclusion, this study identified decreased DNA methylation levels in the promoter regions of IL-17, IL-21, and IL-22 in patients with GD and preliminarily explored their associations with Th17 cell activation and clinical parameters. These findings contribute to a deeper understanding of the epigenetic mechanisms involved in GD and lay a foundation for future research.

## 5 Conclusion

From an epigenetic perspective, this study investigated the aberrant methylation patterns of IL-17, IL-21, and IL-22 genes in GD patients, contributing to a deeper understanding of the role of Th17 cells in GD-related immune dysregulation. These findings may aid in advancing the understanding of GD’s immunopathological mechanisms and provide a scientific basis for potential epigenetic intervention strategies.

## Data Availability

The original contributions presented in the study are included in the article/**Supplementary Material**. Further inquiries can be directed to the corresponding authors.

## Supplementary material

Supplementary Figure 1 DNA methylation patterns of IL17, IL21, and IL22 gene promoters in GD, NGD, and RGD groups compared to normal controls. **(A)** Box plots showing DNA methylation percentages in the IL17, IL21, and IL22 gene promoters in GD patients and NCs. Vertical bars indicate 95% confidence intervals of the mean. **(B)** Heatmap showing methylation levels in the IL17, IL21, and IL22 promoters in GD and NC groups. **(C)** Box plots showing methylation percentages in NGD patients and NCs. **(D)** Heatmap of IL17, IL21, and IL22 promoter methylation levels in NGD and NC groups. **(E)** Box plots showing methylation percentages in RGD patients and NCs. **(F)** Heatmap of IL17, IL21, and IL22 promoter methylation levels in RGD and NC groups. Abbreviations: GD: Graves’ Disease, NGD: Newly Diagnosed Graves’ Disease, RGD: Refractory Graves’ Disease, NC: Normal Control.

Supplementary Figure 2 Univariate logistic regression analysis of DNA methylation sites associated with clinical features. **(A)** Univariate logistic regression analysis of the association between clinical parameters and methylation at two CpG sites located on chromosome 4: chr4_123542199_R (12*99) and chr4_123542549_R (12*49). **(B)** Univariate logistic regression analysis of the association between clinical parameters and methylation at two CpG sites located on chromosome 12: chr12_68647247_R (68*47) and chr12_68647735_R (68*45).
