# Supplementary figures and images for: Th17-associated cytokine gene hypomethylation reflects epigenetic dysregulation in graves’ disease
Source: Front Immunol. 2025 Sep 16;16:1635883. doi: 10.3389/fimmu.2025.1635883 (PMC12479413; doi:10.3389/fimmu.2025.1635883)

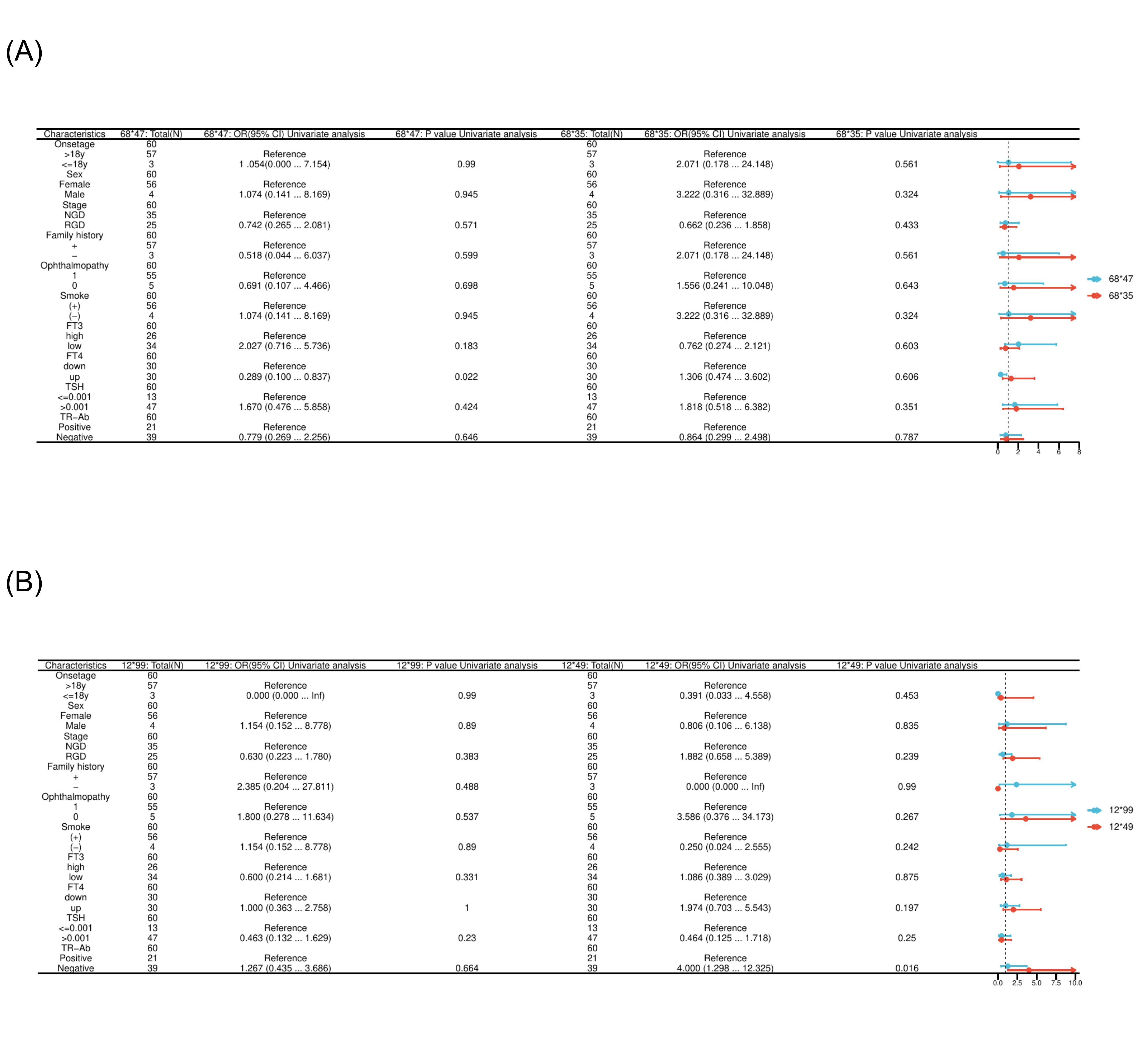

Supplement: Supplementary Figure 2 — Univariate logistic regression analysis of DNA methylation sites associated with clinical features. (A) Univariate logistic regression analysis of the association between clinical parameters and methylation at two CpG sites located on chromosome 4: chr4_123542199_R (12*99) and chr4_123542549_R (12*49). (B) Univariate logistic regression analysis of the association between clinical parameters and methylation at two CpG sites located on chromosome 12: chr12_68647247_R (68*47) and chr12_68647735_R (68*45). [file Image2.tiff]
