# Supplementary material for: Th17-associated cytokine gene hypomethylation reflects epigenetic dysregulation in graves’ disease
Source: Front Immunol. 2025 Sep 16;16:1635883. doi: 10.3389/fimmu.2025.1635883 (PMC12479413; doi:10.3389/fimmu.2025.1635883)
Supplement: Supplementary file 3 [file Table1.docx]

Table S1 Primers and PCR conditions used in pyrosequencing.

| ID | Primers | | PCR conditions |
| --- | --- | --- | --- |
|  | F | R |  |
| IL17-1 | TGTGAGATATATTTAAATAGGATTTTGAAGGA | AAATTCGTACAACTTCCTTTCTAACC | 95℃ for 15 min  (94℃ for 30s, 53.8℃ for 30s, 72℃ for 30s）* 45 cycle  72℃ for 10 min |
| IL17-2 | AATTAGGATTTTATTATTTTAGGTTTTGGAGT | ACTATACTTACTTTTCCCAATTACTTCCT |  |
| IL17-3 | TGTTTTGAGAATTATTATTGAAAGTATGTCG | TCAAAACAAAATTCAAAATAAACCCTACT |  |
| IL17-4 | AGGAAGTAATTGGGAAAAGTAAGTATAGT | ACAACTTCAAATAATTTCCGTAATCCT |  |
| IL17-5 | TGTAGAGGAGGGGTAGTAGGGTTT | TCTTTCCTAACCAAATAAACCCTAACT |  |
| IL17-6 | GGATTACGGAAATTATTTGAAGTTGTATTTTATT | ACAATCTCAACTACCAAAATAACTAATTCT |  |
| IL17-7 | TTATTTATTAGTTAGGGTTTATTTGGTTAGGA | AAAAACCAAAAACTTCCTTCCTCA |  |
| IL17-8 | GGTTGTAGGGATAAAGGGAGTGAGT | CATTCCTCCCTATCCTACTCTACCT |  |
| IL17-9 | GGGAGTTTGAGTAAGTAGTTTTTAGGGT | CCCCCACTAAAATAAATAATAACTCTCA |  |
| IL17-10 | TGTAGAATATGGGATATTAGTTGAGTGT | ACGATTCTTTTCTCTATACTCTAAATAAAATAAAC |  |
| IL17-11 | TTGAGAAGGAATTATTTTTAAGGATTTGAGTT | CACGAAATTTTCTATTAAAAACATATTCCAAC |  |
| IL17-12 | TAAAATATTAAGTTGTTTGGTAGTATGTAGG | ACCAAATATTATAAAAACAATTCCGAAAACT |  |
| IL17-13 | TTTGTTTATTTATATGATGGGAATTTGAGT | ACTAAACAAAACTACTATACTATAAATCAATATC |  |
| IL17-14 | AGAGTATAATTTTTTTGGTAGTTGTATATATG | ACGTATCGCAATAAATTCAAAAATAACAC |  |
| IL17-15 | GATTTTATTGGGGGCGGAAATTT | CCAATAAAATCTTCCCAAAAATCATCGT |  |
| IL21-1 | TGGGGTATTGGTAAGTTTATTATATTAGAGA | ACCTCACCACTATATTAAACAACCCA | 95℃ for 15 min  (94℃ for 30s, 53.8℃ for 30s, 72℃ for 30s）* 45 cycle  72℃ for 10 min |
| IL21-2 | TGATGTATTGGTGGATGGATTATGGA | AAACAAATCAATATTCAACCAAAATACACT |  |
| IL21-3 | GGTATTTTAGGGTTAGGAGTAATGGT | ATCCCGAAAAATTTACATACTAATCGT |  |
| IL21-4 | GATTAGTTTAGTTTTTTTTAGGTTTTTTAAAGTT | TCTAAAACTTAAATTCAAATCAAAATCCTAACA |  |
| IL21-5 | TGAGTAGATTTTGTATTTGTTTTTTTTTTGTAA | TACCACTTAATACCTCCACATTACCTA |  |
| IL21-6 | AATTATCGTGGTTAGTGTTTTTAAGGA | AAAAATTCATACAACATAATCAAAAATTCCTCCC |  |
| IL21-7 | TGGAGGTATTAAGTGGTAATTTTTAGTTT | TCTAAACTATTACGTAACCAACATAAATTAAAA |  |
| IL21-8 | GGAGGAATTTTTGATTATGTTGTATGAATTTTTAT | TCATTCATTCATTCATTTCATTACATACTTT |  |
| IL21-9 | TGGTTACGTAATAGTTTAGAATAAAGTATGGTATT | ATCATCCTCCTTTCTTCTACTTCTC |  |
| IL21-10 | TTTAAATGGGTTAAAAGAGTTTTAGGGA | ACCCATAAATAATTCGTTTTCTCTTAAAAT |  |
| IL21-11 | GAAATGTGTTGTTTATATTTAGAGGAAATAGT | TCTCACTCTACCATCATTTTAAAAATTATCT |  |
| IL21-12 | TGAGTATTAGTAAGTTTGATTTATTTAATTGATGT | CCAACTATTCACCAAAACATCTTTACT |  |
| IL21-13 | GTGAGATTTTGATTGGGAAGGATAGAT | CCATCATAAAAATAAAAAAAAAAACTACCTT |  |
| IL21-14 | AGATGTTTTGGTGAATAGTTGGAAGT | ACAACATATACTTTCATACAAAACTTTTTCT |  |
| IL21-15 | TGGTAAGAATTTAAAATATTTTTGTTAATTTGG | AATCTCTTTTTCTCGACCTCCCCTA |  |
| IL21-16 | AGTGTTTTTATGATTGTTAGAGAGAATAGGT | TCAACGAAATATACCCCATCTACATCT |  |
| IL21-17 | GGTCGAGAAAAAGAGATTAAAGTGTTTTTGTTT | TTCCACTAACTAAATATACGTATACATATACTAAT |  |
| IL21-18 | TGGGGTATATTTCGTTGATTTTATTAATTTTTGT | ACTTAACTAAAAACAAATTATCAATTAAACTACC |  |
| IL21-19 | ATTTATTTTATAAAGATTTTTAGGTTGTAATGGG | AATAACCATCAAACAAATAACAATCCTCTCCA |  |
| IL22-1 | TAGGTTTATTTACGTTATTGTAAATGATAGG | CAAAAAACATAAATTATAAATATTCTCATTAC | 95℃ for 15 min  (94℃ for 30s, 53.8℃ for 30s, 72℃ for 30s）* 45 cycle  72℃ for 10 min |
| IL22-2 | GTATTGTGGAATGGTTAAATTGAGTGAAT | ATTACCAAAAACTAAATAAACAAAAAACAAC |  |
| IL22-3 | GTTGTATTTTTTGGGTAATATTTTTTTATTTC | CACAAACCTAAACAAAATACCACTACAA |  |
| IL22-4 | TGTTTAGTGTTTTTATGTGGTTTCGA | ACAAATACAAATATCAAAATACAAATACCTATTAC |  |
| IL22-5 | TGTGAGGAGAAAAATTTGATTATATAAATTTGT | ACATATATACACACACACACATAAATATCT |  |
| IL22-6 | GGTAACGCGGAATTTTTATATTTTTATATTTTT | ATTCATCTTAAAATTTTACCTAATTCTACACTT |  |
| IL22-7 | TTGGAAATATTATGTTGAGATTTGTGTG | CTTAACTAATCTAATTATTATTTTCTTCCCTT |  |
| IL22-8 | AGGAGATTAGATTTTTAGTATTAGTATTTAT | ATAATAATAACAACTAACAAATCATTCCATTA |  |
| IL22-9 | TGGTTTAGAATTTAGTATGTTTAAAAATGAGA | TCCTATAATAACTAAATAAACATTTTAATCACGA |  |
| IL22-10 | TGAGATAAGAATTTATAAAGTTGGTAGGAAA | TCCCTAAAACGTCACTATTAAAACCCGA |  |
| IL22-11 | TTTTTGAAAAATATGTAGGGTTTAGAAAATTTTTG | AAATATAACATCAACAATTATCTAATTTCCAATAC |  |
| IL22-12 | GGGAAATATTTGTATTTTAAGGTGGAAAGG | ACCGAAACGCTTACCTATTCTAAAAATTCTAC |  |
| IL22-13 | AAGTTAGTATTTTGGGGGTTATAAAAGT | ACAAAACGACCATTACAAACAATTC |  |
| F forward primer, R reverse primer | | | |
