# Supplementary material for: Th17-associated cytokine gene hypomethylation reflects epigenetic dysregulation in graves’ disease
Source: Front Immunol. 2025 Sep 16;16:1635883. doi: 10.3389/fimmu.2025.1635883 (PMC12479413; doi:10.3389/fimmu.2025.1635883)
Supplement: Supplementary file 4 [file Table2.docx]

Table S2: IL17, IL21, and IL22 Promoter CpGs Comparison Between GD and NC Groups

| Gene | CpG Site | 95% CI | p(Mann-Whitney U) |
| --- | --- | --- | --- |
| IL17 | chr6_52049313_F | 0.45 – 1.79 | 0.001 |
| IL17 | chr6_52049431_F | -0.05 – 0.72 | 0.0842 |
| IL17 | chr6_52049562_F | 8.52 – 13.02 | 3.79×10⁻¹² |
| IL17 | chr6_52049644_F | -1.13 – -0.38 | 0.0002 |
| IL17 | chr6_52050133_F | -0.25 – 0.43 | 0.6665 |
| IL17 | chr6_52050190_F | 3.87 – 7.30 | 2.12×10⁻⁸ |
| IL17 | chr6_52050263_F | 0.95 – 2.83 | 0.0003 |
| IL17 | chr6_52050401_F | -2.43 – 0.18 | 0.0927 |
| IL17 | chr6_52050597_F | 2.40 – 5.19 | 3.64×10⁻⁷ |
| IL17 | chr6_52050745_F | 0.36 – 1.44 | 0.001 |
| IL17 | chr6_52051062_F | -0.18 – 1.10 | 0.1466 |
| IL17 | chr6_52051103_F | 1.18 – 2.12 | 1.35×10⁻⁹ |
| IL17 | chr6_52051108_F | 2.24 – 4.56 | 8.84×10⁻⁷ |
| IL17 | chr6_52051113_F | 1.43 – 2.73 | 4.89×10⁻⁹ |
| IL17 | chr6_52051156_F | 1.64 – 2.62 | 2.18×10⁻¹¹ |
| IL17 | chr6_52051162_F | 1.02 – 1.93 | 1.39×10⁻⁸ |
| IL21 | chr4_123542199_R | 9.35 – 14.46 | 2.16×10⁻¹² |
| IL21 | chr4_123542356_R | 12.80 – 19.09 | 7.48×10⁻¹² |
| IL21 | chr4_123542401_R | 3.12 – 5.48 | 9.63×10⁻¹¹ |
| IL21 | chr4_123542549_R | 14.68 – 21.16 | 3.37×10⁻¹⁴ |
| IL21 | chr4_123543093_R | 8.38 – 14.09 | 9.07×10⁻¹² |
| IL21 | chr4_123543180_R | 1.21 – 2.76 | 2.61×10⁻⁶ |
| IL21 | chr4_123543435_R | -0.78 – 1.08 | 0.7903 |
| IL21 | chr4_123543739_R | 5.43 – 9.84 | 2.04×10⁻⁹ |
| IL21 | chr4_123543922_R | 1.93 – 5.09 | 1.42×10⁻⁵ |
| IL21 | chr4_123543942_R | 3.02 – 6.89 | 2.01×10⁻⁶ |
| IL22 | chr12_68647247_R | 3.19 – 5.08 | 1.87×10⁻¹¹ |
| IL22 | chr12_68647281_R | 1.37 – 2.66 | 2.81×10⁻⁸ |
| IL22 | chr12_68647290_R | -1.63 – -0.47 | 0.0004 |
| IL22 | chr12_68647352_R | -0.12 – 0.50 | 0.2189 |
| IL22 | chr12_68647357_R | 0.39 – 0.97 | 3.51×10⁻⁶ |
| IL22 | chr12_68647388_R | 1.02 – 1.76 | 4.28×10⁻¹⁰ |
| IL22 | chr12_68647499_R | 9.98 – 13.98 | 7.58×10⁻¹³ |
| IL22 | chr12_68647575_R | 2.59 – 4.61 | 9.63×10⁻¹¹ |
| IL22 | chr12_68647591_R | 2.09 – 3.31 | 2.91×10⁻¹⁴ |
| IL22 | chr12_68647735_R | 4.96 – 7.38 | 7.73×10⁻¹³ |
| IL22 | chr12_68648357_R | -0.48 – 0.07 | 0.1785 |
| IL22 | chr12_68648359_R | -0.20 – 0.30 | 0.7675 |
| IL22 | chr12_68648412_R | -0.04 – 0.74 | 0.0739 |
| IL22 | chr12_68648630_R | 0.30 – 0.88 | 0.0002 |
| IL22 | chr12_68648813_R | 1.14– 2.15 | 4.65×10⁻⁸ |
| IL22 | chr12_68648921_R | 0.46 – 1.09 | 5.30×10⁻⁶ |
| IL22 | chr12_68649043_R | 0.06 – 0.47 | 0.0172 |

95% CI 95% Confidence Interval, F forward primer, R reverse primer
