# Supplementary material for: Th17-associated cytokine gene hypomethylation reflects epigenetic dysregulation in graves’ disease
Source: Front Immunol. 2025 Sep 16;16:1635883. doi: 10.3389/fimmu.2025.1635883 (PMC12479413; doi:10.3389/fimmu.2025.1635883)
Supplement: Supplementary file 5 [file Table3.docx]

| Table S3 Risk Assessment of four CpGs using logistic regression analysis | | | | | | |
| --- | --- | --- | --- | --- | --- | --- |
| CpG Site | Characteristics | Total(N) | OR(95% CI) Univariate analysis | p value Univariate analysis | OR(95% CI) Multivariate analysis | p value Multivariate analysis |
| chr4_123542199_R | Onset age | 60 |  |  |  |  |
|  | <=18y | 57 | Reference |  |  |  |
|  | >18y | 3 | 0.00 (0.00 – Inf) | 0.99 |  |  |
|  | Sex | 60 |  |  |  |  |
|  | Female | 56 | Reference |  |  |  |
|  | Male | 4 | 1.15 (0.15 – 8.78) | 0.89 |  |  |
|  | Stage | 60 |  |  |  |  |
|  | Normal | 35 | Reference |  |  |  |
|  | I/II/III | 25 | 0.63 (0.22 – 1.78) | 0.38 |  |  |
|  | Family history | 60 |  |  |  |  |
|  | (+) | 57 | Reference |  |  |  |
|  | (-) | 3 | 2.39 (0.20 – 27.81) | 0.49 |  |  |
|  | Ophthalmopathy | 60 |  |  |  |  |
|  | (+) | 55 | Reference |  |  |  |
|  | (-) | 5 | 1.80 (0.28 – 11.63) | 0.54 |  |  |
|  | Smoke | 60 |  |  |  |  |
|  | (+) | 56 | Reference |  |  |  |
|  | (-) | 4 | 1.15 (0.15 – 8.78) | 0.89 |  |  |
|  | FT3 | 60 |  |  |  |  |
|  | Upper 50% | 26 | Reference |  |  |  |
|  | Lower 50% | 34 | 0.60 (0.21 – 1.68) | 0.33 |  |  |
|  | FT4 | 60 |  |  |  |  |
|  | Upper 50% | 30 | Reference |  |  |  |
|  | Lower 50% | 30 | 1.00 (0.36 – 2.76) | 1.00 |  |  |
|  | TSH | 60 |  |  |  |  |
|  | > 0.001 | 13 | Reference |  |  |  |
|  | <= 0.001 | 47 | 0.46 (0.13 – 1.63) | 0.23 |  |  |
|  | TR-Ab（0-1.5） | 60 |  |  |  |  |
|  | <= 1.5 | 21 | Reference |  |  |  |
|  | > 1.5 | 39 | 1.27 (0.44 – 3.69) | 0.66 |  |  |
| chr4_123542549_R | Onset age | 60 |  |  |  |  |
|  | <=18y | 57 | Reference |  |  |  |
|  | >18y | 3 | 0.39 (0.03 – 4.56) | 0.45 |  |  |
|  | Sex | 60 |  |  |  |  |
|  | Female | 56 | Reference |  |  |  |
|  | Male | 4 | 0.81 (0.11 – 6.14) | 0.84 |  |  |
|  | Stage | 60 |  |  |  |  |
|  | Normal | 35 | Reference |  |  |  |
|  | I/II/III | 25 | 1.88 (0.66 – 5.39) | 0.24 |  |  |
|  | Family history | 60 |  |  |  |  |
|  | (+) | 57 | Reference |  |  |  |
|  | (-) | 3 | 0.00 (0.00 – Inf) | 0.99 |  |  |
|  | Ophthalmopathy | 60 |  |  |  |  |
|  | (+) | 55 | Reference |  |  |  |
|  | (-) | 5 | 3.59 (0.38 – 34.17) | 0.27 |  |  |
|  | Smoke | 60 |  |  |  |  |
|  | (+) | 56 | Reference |  |  |  |
|  | (-) | 4 | 0.25 (0.02 – 2.56) | 0.24 |  |  |
|  | FT3 | 60 |  |  |  |  |
|  | Upper 50% | 26 | Reference |  |  |  |
|  | Lower 50% | 34 | 1.09 (0.39 – 3.03) | 0.88 |  |  |
|  | FT4 | 60 |  |  |  |  |
|  | Upper 50% | 30 | Reference |  |  |  |
|  | Lower 50% | 30 | 1.98 (0.70 – 5.54) | 0.20 |  |  |
|  | TSH | 60 |  |  |  |  |
|  | > 0.001 | 13 | Reference |  |  |  |
|  | <= 0.001 | 47 | 0.46 (0.13 – 1.72) | 0.25 |  |  |
|  | TR-Ab（0-1.5） | 60 |  |  |  |  |
|  | <= 1.5 | 21 | Reference |  | Reference |  |
|  | > 1.5 | 39 | 4.00 (1.30 – 12.33) | 0.02 | 4.00 (1.30 – 12.33) | 0.02 |
| chr12_68647247_R | Onset age | 60 |  |  |  |  |
|  | <=18y | 57 | Reference |  |  |  |
|  | >18y | 3 | 18661237.87 (0.00 – Inf) | 0.99 |  |  |
|  | Sex | 60 |  |  |  |  |
|  | Female | 56 | Reference |  |  |  |
|  | Male | 4 | 1.07 (0.14 – 8.17) | 0.95 |  |  |
|  | Stage | 60 |  |  |  |  |
|  | Normal | 35 | Reference |  |  |  |
|  | I/II/III | 25 | 0.74 (0.27 – 2.08) | 0.57 |  |  |
|  | Family history | 60 |  |  |  |  |
|  | (+) | 57 | Reference |  |  |  |
|  | (-) | 3 | 0.52 (0.04 – 6.04) | 0.6 |  |  |
|  | Ophthalmopathy | 60 |  |  |  |  |
|  | (+) | 55 | Reference |  |  |  |
|  | (-) | 5 | 0.69 (0.11 – 4.47) | 0.70 |  |  |
|  | Smoke | 60 |  |  |  |  |
|  | (+) | 56 | Reference |  |  |  |
|  | (-) | 4 | 1.07 (0.14 – 8.17) | 0.95 |  |  |
|  | FT3 | 60 |  |  |  |  |
|  | Upper 50% | 26 | Reference |  |  |  |
|  | Lower 50% | 34 | 2.03 (0.72 – 5.74) | 0.18 |  |  |
|  | FT4 | 60 |  |  |  |  |
|  | Upper 50% | 30 | Reference |  | Reference |  |
|  | Lower 50% | 30 | 0.29 (0.10 – 0.84) | 0.02 | 0.29 (0.10 – 0.84) | 0.02 |
|  | TSH | 60 |  |  |  |  |
|  | > 0.001 | 13 | Reference |  |  |  |
|  | <= 0.001 | 47 | 1.67 (0.48 – 5.86) | 0.42 |  |  |
|  | TR-Ab（0-1.5） | 60 |  |  |  |  |
|  | <= 1.5 | 21 | Reference |  |  |  |
|  | > 1.5 | 39 | 0.78 (0.27 – 2.26) | 0.65 |  |  |
| chr12_68647735_R | Onset age | 60 |  |  |  |  |
|  | <=18y | 57 | Reference |  |  |  |
|  | >18y | 3 | 2.07 (0.18 – 24.15) | 0.56 |  |  |
|  | Sex | 60 |  |  |  |  |
|  | Female | 56 | Reference |  |  |  |
|  | Male | 4 | 3.22 (0.32 – 32.89) | 0.32 |  |  |
|  | Stage | 60 |  |  |  |  |
|  | Normal | 35 | Reference |  |  |  |
|  | I/II/III | 25 | 0.66 (0.24 – 1.86) | 0.43 |  |  |
|  | Family history | 60 |  |  |  |  |
|  | (+) | 57 | Reference |  |  |  |
|  | (-) | 3 | 2.07 (0.18 – 24.15) | 0.56 |  |  |
|  | Ophthalmopathy | 60 |  |  |  |  |
|  | (+) | 55 | Reference |  |  |  |
|  | (-) | 5 | 1.56 (0.24 – 10.05) | 0.64 |  |  |
|  | Smoke | 60 |  |  |  |  |
|  | (+) | 56 | Reference |  |  |  |
|  | (-) | 4 | 3.22 (0.32 – 32.89) | 0.32 |  |  |
|  | FT3 | 60 |  |  |  |  |
|  | Upper 50% | 26 | Reference |  |  |  |
|  | Lower 50% | 34 | 0.76 (0.27 – 2.12) | 0.6 |  |  |
|  | FT4 | 60 |  |  |  |  |
|  | Upper 50% | 30 | Reference |  |  |  |
|  | Lower 50% | 30 | 1.31 (0.47 – 3.60) | 0.61 |  |  |
|  | TSH | 60 |  |  |  |  |
|  | > 0.001 | 13 | Reference |  |  |  |
|  | <= 0.001 | 47 | 1.82 (0.52 – 6.38) | 0.35 |  |  |
|  | TR-Ab（0-1.5） | 60 |  |  |  |  |
|  | <= 1.5 | 21 | Reference |  |  |  |
|  | > 1.5 | 39 | 0.86 (0.30 – 2.50) | 0.79 |  |  |
| OR Odds Ratio, 95% CI 95% Confidence Interval (95% CI) , Normal, No thyroid enlargement, thyroid is not palpable, or not visible on imaging; I, Mild thyroid enlargement, palpable only when swallowing, or detectable by imaging; II, Moderate thyroid enlargement, palpable at rest, and clearly visible on imaging; III, Severe thyroid enlargement, visibly affecting the neck, often forming a visible goiter, and affecting swallowing and breathing. (+), indicates the presence of the condition (e.g., family history, ophthalmopathy, smoking); (-), indicates the absence of the condition. FT3, Free Triiodothyronine; FT4, Free Thyroxine; TSH, Thyrotropin (Thyroid-Stimulating Hormone); TRAb, TSH Receptor Antibody. | | | | | | |
