# Supplementary material for: Th17-associated cytokine gene hypomethylation reflects epigenetic dysregulation in graves’ disease
Source: Front Immunol. 2025 Sep 16;16:1635883. doi: 10.3389/fimmu.2025.1635883 (PMC12479413; doi:10.3389/fimmu.2025.1635883)
Supplement: Supplementary file 6 [file Table4.docx]

Table S4: Variable assignment table.

| Variables | Assignment |
| --- | --- |
| Onset age | 0 <=18y, 1 >18y |
| Sex | 0 = male, 1 = female |
| Stage | 0 = Normal, 1 = I/II/III |
| Family history | 0 = (+), 1 = (-) |
| Ophthalmopathy | 0 = (+), 1 = (-) |
| Smoke | 0 = (+), 1 = (-) |
| FT3 | 0 = Upper 50%, 1 = Lower 50% |
| FT4 | 0 = Upper 50%, 1 = Lower 50% |
| TSH | 0 > 0.001, 1 <= 0.001 |
| TR-Ab（0-1.5） | 0 <= 1.5, 1 > 1.5 |

Normal, No thyroid enlargement, thyroid is not palpable, or not visible on imaging; I, Mild thyroid enlargement, palpable only when swallowing, or detectable by imaging; II, Moderate thyroid enlargement, palpable at rest, and clearly visible on imaging; III, Severe thyroid enlargement, visibly affecting the neck, often forming a visible goiter, and affecting swallowing and breathing. (+), indicates the presence of the condition (e.g., family history, ophthalmopathy, smoking); (-), indicates the absence of the condition. FT3, Free Triiodothyronine; FT4, Free Thyroxine; TSH, Thyrotropin (Thyroid-Stimulating Hormone); TRAb, TSH Receptor Antibody.
